# Establishing a Novel Gene Signature Related to Histone Modifications for Predicting Prognosis in Lung Adenocarcinoma

**DOI:** 10.1155/2022/8802573

**Published:** 2022-09-23

**Authors:** Mengfeng Liu, Xiran Yu, Shidong Xu, Changfa Qu

**Affiliations:** Department of Thoracic Surgery, Harbin Medical University Cancer Hospital, Harbin 150081, China

## Abstract

**Background:**

Epigenetic modifications have been revealed to play an important role in tumorigenesis and tumor development. This study aims to analyze the role of histone modifications and the prognostic values of histone modifications in lung adenocarcinoma (LUAD). The promoters and enhancers of protein encoding genes (PCGs) were the regions of enriched histone modifications.

**Methods:**

Expression profiles and clinical information of LUAD samples were downloaded from the Cancer Genome Atlas (TCGA) and Gene Expression Omnibus (GEO) databases. Histone modification data of LUAD cell lines were downloaded from Encyclopedia of DNA Elements (ENCODE) database. Limma *R* package was used to identify differentially expressed PCGs. To identify molecular subtypes, consensus clustering was conducted based on the expression of dysregulated PCGs with abnormal histone modifications. Univariate Cox regression analysis and stepwise Akaike information criterion (stepAIC) were utilized to establish a prognostic model.

**Results:**

We identified a total of 699 epigenetic dysregulated genes with 122 of them significantly correlating with LUAD prognosis. We constructed three molecular subtypes (C1, C2, and C3) based on the 122 prognostic genes. C2 had the longest overall survival while C1 had the worst prognosis. In addition, three subtypes had differential immune infiltration and the response to immune checkpoint inhibitors. Moreover, we identified a risk model containing 5 epi-PCGs that had favorable performance to predict prognosis in different datasets.

**Conclusions:**

This study further supported the critical histone modifications in LUAD development. Three subtypes may provide guidance for the immunotherapy of LUAD patients. Importantly, the prognostic model had great potential to predict LUAD prognosis.

## 1. Introduction

Lung cancer is the second most diagnosed cancer worldwide, which contributes a lot to the cancer burden globally [1]. Lung cancer as a leading cause of cancer mortality has a less than 20% survival rate [2]. According to the global cancer statistics, a ratio of 39/100,000 has occurred in men that ranking first, and 18.2/100,000 in women [1]. Lung adenocarcinoma (LUAD) is the most common histological type of nonsmall cell lung cancer (NSCLC), composing approximately 40% of malignant lung cancer cases [3]. In the Chinese population, the proportion of LUAD patients in lung cancer increased largely from 34.4% to 59.3% in 2003–2012, especially a massive increase in the female population [4].

Although smoking is considered as the most common risk factor, the number of diagnosed female patients has been rising obviously in the recent decades. The increasing diagnostic rate of nonsmoking individuals in LUAD highlights the importance of other risk factors such as passive smoking, cooking fumes, air pollution, and gene susceptibility [4, 5]. Large-scale genomic analysis on LUAD revealed that KRAS, EGFR, CDKN2A, TP53, and KEAP1 were commonly mutated in LUAD patients [2]. A distinct genomic landscape is characterized between smokers and nonsmokers, with the former presenting a higher mutation frequency [2]. These differential mutations induced a differential tumor microenvironment (TME), accounting for the different response of patients to targeted therapies such as nivolumab [6], pembrolizumab [7], and osimertinib [8]. About 70% patients are already advanced or metastatic when diagnosing as lung cancer [5]. Only a small part of patients can benefit much from traditional therapies such as chemotherapy and radiotherapy. Although some targeted molecules have been developed to function a positive effect on LUAD patients, still a large fraction of patients are negatively responsive possibly due to disadvantageous TME and distinct gene mutation subtypes.

Epigenetic effect has been identified as a critical factor in modulating gene expression, which is considered to have prognostic value in many cancers [9]. Histone modifications are the most common modifications in DNA, and are significantly associated with NSCLC survival and metastasis [10]. In this study, we characterized the effects of histone acetylation (ac) and methylation (me) (H3K9me3, H3K27me3, H3K27ac, and H3K4me3) on LUAD patients, and developed novel molecular subtypes and a 5-gene signature based on these modifications. Importantly, we emphasized the relation between histone modifications and cancer development, and the role of these modifications in guiding targeted therapies for LUAD patients.

## 2. Materials and Methods

### 2.1. Data Information of Gene Expression Profiles and Histone Modification

From TCGA database and GEO database, gene expression data with LUAD clinical information were downloaded in September 1, 2021. For TCGA-LUAD dataset, we removed the samples with no survival status and overall survival. A total of 500 LUAD samples were remained in TCGA-LUAD dataset. For five GSE cohorts (GSE19188, GSE30219, GSE31210, GSE37745, and GSE50081) from GEO, expression data were combined through “removeBatchEffect” function in limma *R* package [11]. PCA showed that batch effect was eliminated after processing (Supplementary Figure S1). The detailed information of screened LUAD samples in TCGA and GSE cohorts was listed in Supplementary Table S1.

The replicated narrowPeak data (hg38) of LUAD cell line (PC-9) and normal lung fibroblasts (AG04450) containing four types of histone modification (H3K27me3, H3K9me3, H3K4me3, and H3K27ac) was downloaded from Encyclopedia of DNA Elements database in September 1, 2021.

### 2.2. Identification of Protein-Coding Genes (PCGs) with Dysregulated Epigenetic Modifications

Limma *R* package was employed to filter differentially expressed PCGs with FDR <0.05 and |fold change (FC)| > 1.5. Then based on the peak location of histone modification, we screened differential peaks with *P* < 0.05. Using GTF file (GRCh38.p13) from GENCODE database, we annotated the corresponding genes of differential peaks. The data of enhancers were obtained from FANTOM5 database [12]. The location of promoters were determined in the region of 2 kb and 0.5 kb in transcriptional start site (TSS) upstream and downstream, respectively, and ChIPseeker *R* package was used to identify promoters [13]. PCGs with dysregulated histone modifications were defined as epi-PCGs following with two criterions: (1) PCGs were differentially expressed between normal and tumor samples; (2) dysregulated histone modification was existed at least in one location (promoters or enhancers). PCGs without differential expression or dysregulated histone modification were defined as non-epi-PCGs.

### 2.3. Gene Enrichment Analysis

SGSEA is a popular method with ability to calculate normalized enrichment score of a gene set for each sample [14, 15]. We performed the algorithm of “ssGSEA” in GSVA *R* package [16] to calculate the enrichment score of eight types of epi-PCGs (four types of dysregulated histone modifications in enhancers and promoters, respectively) in normal tissues and tumor tissues. Wilcoxon rank test compared the difference of enrichment score in tumor and normal tissues. The enrichment score of KEGG pathways was also calculated via ssGSEA. The annotation for significantly enriched KEGG pathways and gene ontology terms was outputted through clusterProfiler *R* package [17]. *P* < 0.05 was set as a cut-off to detect enriched pathways and terms. Only the top 10 were visualized.

### 2.4. Molecular Subtyping Based on Epi-PCGs

Univariate Cox regression analysis was performed to screen epi-PCGs associated with prognosis (*P* < 0.05) in TCGA-LUAD and GEO datasets. The intersected epi-PCGs between two datasets were used as a basis for unsupervised consensus clustering using ConsensusClusterPlus *R* package [18]. The optimal cluster number *k* (*k* was tested from 2 to 10) was confirmed through cumulative distribution function (CDF) and Kaplan‒Meier survival plot.

### 2.5. Assessment of TME

Three gene signatures of Th1/IFN-*γ*, cytolytic (CYT) score, angiogenesis score were obtained from previous studies [19–21], and the enrichment score of them was calculated by ssGSEA [15]. From Danilova et al. study, 47 immune checkpoints were obtained [19]. CIBERSORT estimates the fraction of 22 immune cells in a mixture [22], and it was applied in our study to evaluate the immune infiltration of thee subtypes. In addition, another 28 immune cells were obtained from Charoentong et al. [23], and their enrichment score of three subtypes was calculated by ssGSEA. ANOVA was conducted among three subtypes.

### 2.6. Prediction of Response to Immunotherapy

TIDE analysis was applied to predict the response to immune checkpoint blockade [24]. By evaluating the score of three aspects including TIDE, T cell exclusion, and T cell dysfunction, the sensitivity to immunotherapy could be estimated. Higher TIDE score represents higher possibility of immune escape and lower sensitivity to immunotherapy. Higher score T cell dysfunction indicates worse T cell function, and higher T cell exclusion indicates lower infiltration of T cells. Both of them were negatively with the sensitivity to immunotherapy. ANOVA was conducted among three subtypes.

### 2.7. Establishment and Validation of a Prognostic Model

Firstly, TCGA-LUAD samples were divided into training group and test group with a ratio of 3 : 2 through 100 times random sampling. The optimal training and test groups were confirmed according to the similar clinical information and gene expression profiles between two groups. No difference between two groups was shown in Chi-square test (*P* > 0.05, Supplementary Table S2). In the training group, univariate Cox regression analysis was performed on 122 epi-PCGs (using for consensus clustering) to identify significant prognosis-associated genes (*P* < 0.05). LASSO Cox regression in glmnet *R* package [25] and stepwise Akaike information criterion (stepAIC) in MASS *R* package [26] was here to decrease prognostic genes and construct a prognosis model. The model was defined by risk score = Σ (coefficient(i)^*∗*^expression(i)), where LASSO coefficient was used and *i* present genes.

Risk score was converted to z-score. To classify samples into two risk groups, and z-score = 0 was set as a cut-off. ROC curve was characterized to evaluate the effectiveness of the model through timeROC *R* package [27]. The robustness of the prognostic model was verified in independent validation and test groups by using ROC and survival analysis. Univariate and multivariate Cox regression analyses demonstrated the risk score as an independent indicator, comparing with other clinical features.

### 2.8. Application of the Prognostic Model in Clinical

Constructing a nomogram is a popular method to visualize a prognostic model for clinical use. Analysis on multivariate Cox regression analysis determined that risk factors of *P* < 0.05 were used as an input to construct a nomogram through rms *R* package. The 1-year, 3-year, and 5-year death rates could be indicated from the total points. DCA in ggplot2 R package was applied to evaluate the efficiency of the nomogram by comparing with other indicators [28]. DCA predicts the relative benefits to manage a true positive case and harms to treat a false positive case judging by threshold possibility.

### 2.9. Comparison with Other Prognostic Models

Five prognostic models for LUAD were selected from previous studies, including a 7-gene signature from Al-Dherasi et al. [29], an 8-gene signature from Li et al. [30], a 3-gene signature from Liu et al. [31], a 4-gene signature from Sun et al. [32], and a 12-gene signature from Xue et al. [33]. To fairly compare their efficiency, the same dataset (TCGA-LUAD) was used, and ROC analysis was performed individually for five signatures.

### 2.10. Statistical Analysis

R (v4.1.0) software was used in this study to conduct all statistical analysis. Parameters not showing were default. Wilcoxon test was for two group analysis, and ANOVA was for three-group analysis. Log-rank test was conducted in survival analysis and Cox regression analysis. *P* < 0.05 was considered as significant. Ns, no significance. ^∗∗∗∗^*P* < 0.0001, ^∗∗∗^*P* < 0.001, ^∗∗^*P* < 0.01, and ^∗^*P* < 0.05.

## 3. Results

### 3.1. The Number and Length of PCG Transcripts and Exons may be Affect by Histone Modifications

A line of studies have demonstrated that the histone modifications in enhancers and promoters can regulate the expression of a gene and therefore results in cascade effects in oncogenic pathways. We thus focus on the effect of histone modifications in enhancers and promoters to tumorigenesis in LUAD. Firstly, in TCGA-LUAD dataset, we identified the differentially expressed PCGs in tumor and normal lung tissues. A total of 5454 differentially expressed PCGs were identified (FDR <0.05). Within 5454 PCGs, we further compared the histone modifications of these PCGs in tumor samples with those in TCGA-LUAD dataset. Groups of epi-PCG and non-epi-PCG were defined to represent dysregulated and normal histone modifications, respectively. Finally, 699 epi-PCGs were screened counting for a proportion of 3.58% in 5454 PCGs.

**Figure 1:**
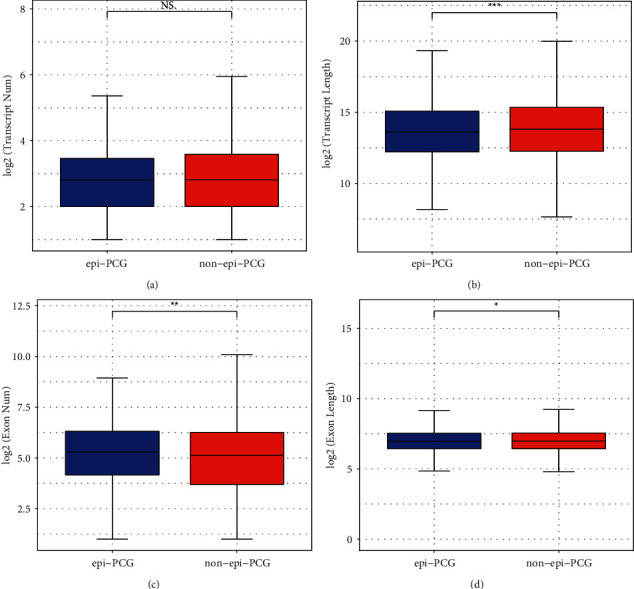
The genomic features of epi-PCGs and non-epi-PCGs. (a‒b) the number and length of transcripts in epi-PCGs and non-epi-PCGs. (c‒d) the number and length of exons in epi-PCGs and non-epi-PCGs. Wilcoxon test was performed. NS, no significance. ^∗^*P* < 0.05, ^∗∗^*P* < 0.01, and ^∗∗∗^*P* < 0.001.

Then we tried to clarify the relation between dysregulated histone modifications and the activity of gene expression or the length of gene transcripts. The result showed no difference on transcript numbers between epi-PCG and non-epi-PCG groups (*P* > 0.05, Figure 1(a)). However, significant difference was observed on the length of transcript between two groups (*P* < 0.001, Figure 1(b)). In regard to exons, we also calculated their number and length, and both of which manifested significant difference between two groups (*P* < 0.05, Figures 1(c) and 1(d)). Epi-PCG group had more exon numbers than non-epi-PCG group, while the exon length in epi-PCG group was a few less than non-epi-PCG group.

**Figure 2:**
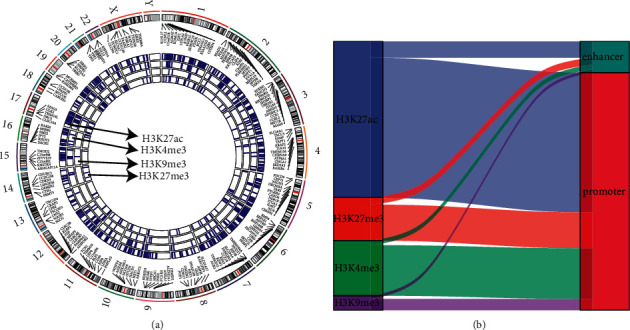
The genome landscape of histone modifications. (a) The distribution of histone modifications of epi-PCGs in the whole genome. (b) The distribution of H3K9me3, H3K27me3, H3K27ac, and H3K4me3 in epi-PCGs.

In addition, we visualized the distribution of 699 epi-PCGs in a genome map. It could be apparently observed that four major types of histone modifications consisted the majority histone dysregulations including H3K9me3, H3K27me3, H3K27ac, and H3K4me3 (Figure 2(a)). Except for Y chromosome, other 23 chromosomes were all responsible for the contribution of dysregulated histone modifications. Furthermore, these dysregulated modifications with H3K27ac as the most affected were accumulated in promoters with a small fraction in enhancers (Figure 2(b)). We suspected that the dysregulation of epi-PCGs may be largely induced by the dysregulated H3K27ac in their promoters.

### 3.2. Enriched Pathways and GO terms of Epi-PCGs

**Figure 3:**
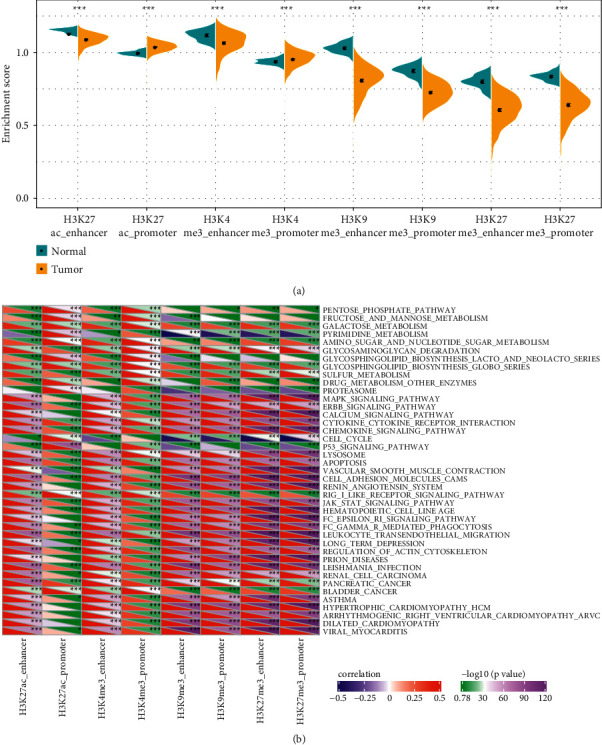
Functional analysis of epi-PCGs. (a) The expression of eight types of epi-PCGs in normal and tumor samples. Student's *t*-test was performed. (b) Pearson correlation analysis between enrichment score of epi-PCGs and KEGG pathways. ^∗∗∗^*P* < 0.0001.

To investigate the role of epi-PCGs in the tumorigenesis, we calculated the enrichment score of each dysregulated histone modification per sample. In all four types of histone modifications in enhancers and promoters, significantly differential enrichment was presented (*P* < 0.001, Figure 3(a)). Besides H3K27ac_promoter and H3K4me3_promoter were more enriched in tumor samples, the enrichment of other six modifications were markedly decreased in tumor samples, indicating that these six modifications may serve as suppressive roles in tumorigenesis (Figure 3(a)).

To characterize the function of these dysregulated epi-PCGs, we performed ssGSEA to evaluate the enrichment of each KEGG pathway in TCGA-LUAD samples. Pearson correlation analysis revealed the 41 most significantly enriched and correlated pathways in eight types of histone modifications (*P* < 0.05, Figure 3(b)). Oncogenic pathways such as p53 signaling pathway, ERBB signaling pathway, chemokine signaling pathway, JAK-STAT signaling pathway, and cell adhesion molecules, metabolism-related pathways such as galactose metabolism, pyrimidine metabolism, pyrimidine metabolism, and drug metabolism other enzymes, and immune-related pathways such as cytokine-cytokine receptor interaction, chemokine signaling pathway, and leukocyte transendothelial migration were enriched and all significantly correlated with eight modifications. The majority of pathways were found to be positively related to the enrichment of four histone modifications. However, two pathways including pyrimidine metabolism and cell cycle exhibited an obviously negative correlation.

Furthermore, we used clusterProfiler to annotate GO terms and KEGG pathways, and obtained the coincident result. The top 10 enriched GO terms and KEGG pathways were visualized (*P* < 0.05, Supplementary Figure S2). Immune-related terms such as cytokine secretion, neutrophil-related immune response and myeloid leukocyte mediated immunity were enriched (Supplementary Figures S2A–S2C). The pathway of cell adhesion molecules was also annotated (Supplementary Figure S2D). The abovementioned results supported that histone modifications contributed an important role in LUAD tumorigenesis.

### 3.3. Molecular Subtyping Based on Epi-PCGs

**Figure 4:**
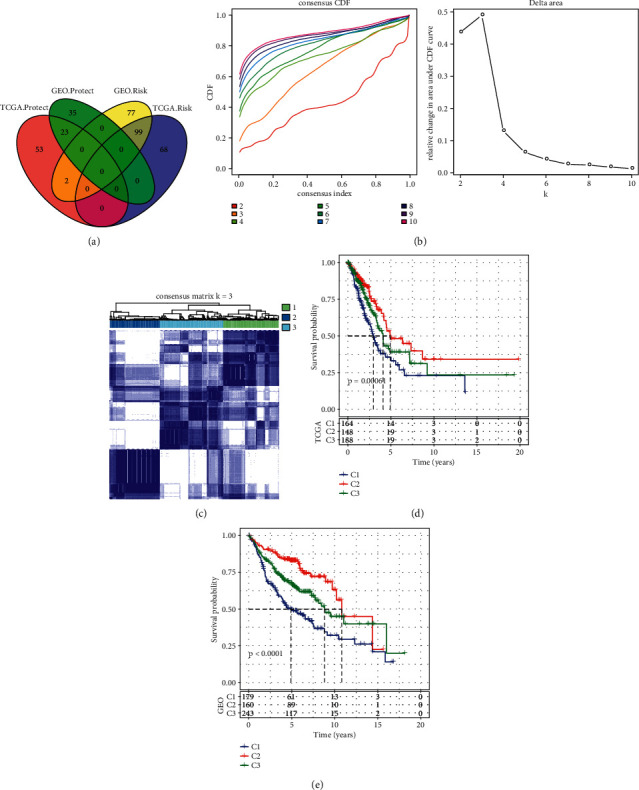
Construction of molecular subtypes based on epi-PCGs. (a) Venn plot for identifying the intersected epi-PCGs associated with prognosis between TCGA-LUAD and GEO datasets. Protect means the positive correlation between the expression of epi-PCGs and survival, and risk means the converse one. (b) CDF curve of cluster number *k* = 2 to 10 in unsupervised consensus clustering. (c) Consensus matrix when *k* = 3. (d‒e) Kaplan‒Meier survival analysis of three molecular subtypes in TCGA-LUAD (d) and GEO (e) datasets. Log-rank test was conducted.

Next we tried to construct a molecular subtyping system for LUAD based on epi-PCGs. Before that, we firstly assess the association between 699 epi-PCGs and LUAD prognosis in both TCGA-LUAD and GEO datasets through univariate Cox regression analysis. As a result, 122 epi-PCGs were identified, and their expression in tumor samples to normal samples were compared with significant difference (Supplementary Figure S3). Then 122 epi-PCGs were selected as candidates to construct molecular subtypes (Figure 4(a)). Unsupervised consensus clustering was conducted through ConsensusClusterPlus, and cluster number *k* from 2 to 10 was evaluated by CDF (Figure 4(b)). Finally, we determined *k* = 3 as the indicator to define three molecular subtypes (C1, C2, and C3, Figure 4(c)). Kaplan‒Meier survival analysis presented the differential prognosis among three molecular subtypes in both TCGA-LUAD and GEO datasets, where the longest survival was shown in C2 subtype and the worst prognosis was shown in C1 subtype (*P*=0.00061 and *P* < 0.0001, respectively, Figure 4(d)).

### 3.4. Distinct TME among Three Subtypes

TME is a critical feature of tumor progression and immune response to against tumor cells. We attempted to unravel if there was a difference of TME among three subtypes according to a series of indicators such as the distribution of immune cells, chemokines, chemokine receptors, tumor-related signatures, and immune checkpoints.

**Figure 5:**
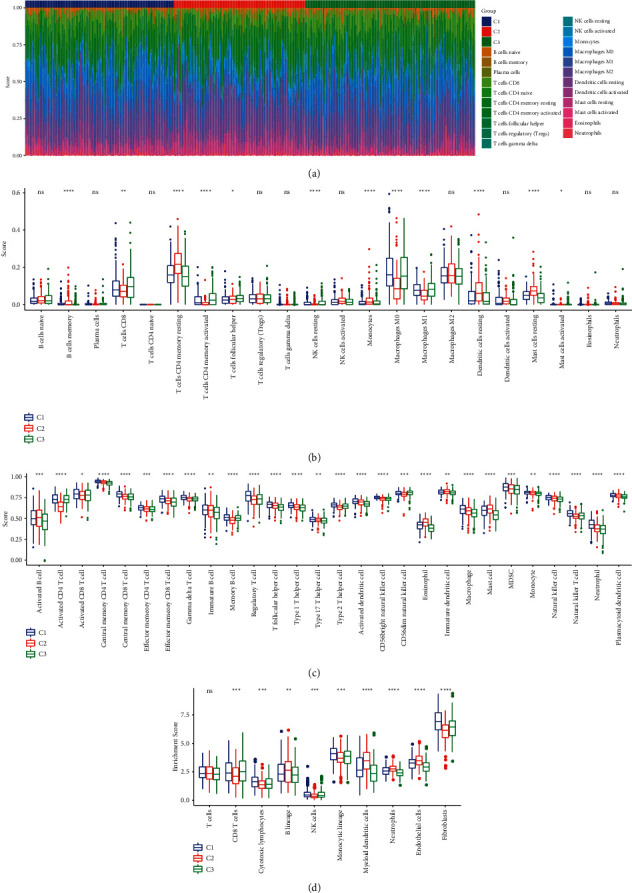
The distribution of immune-related cells in TME. (a) A heatmap presenting the distribution of 22 immune cells in three subtypes. (b) Enrichment score of 22 immune cells in three subtypes assessed by CIBERSORT. (c) Enrichment score of 28 immune cells in three subtypes assessed by ssGSEA. (d) Enrichment score of 10 immune cells analyzed by MCP-counter analysis. ANOVA test was conducted.NS, no significance. ^∗^*P* < 0.05, ^∗∗^*P* < 0.01, ^∗∗∗^*P* < 0.001, and ^∗∗∗∗^*P* < 0.0001.

We performed CIBERSORT to characterize the estimated fraction of 22 immune cells in tumor tissues, and observed that 12 of 22 immune cells were differentially enriched among three subtypes (*P* < 0.05, Figures 5(a) and 5(b)). C3 subtype had a higher proportion of CD8 T cells, but significantly higher macrophages were also shown simultaneously (Figure 5(b)). In addition, we obtained the signatures of 28 immune cells from the previous study, and analyzed their proportions in TCGA-LUAD dataset. Similarly, distinct distributions of all 28 immune cells from ssGSEA results were exhibited among three subtypes (*P* < 0.05, Figure 5(c)). Moreover, we applied MCP-counter to evaluate the immune infiltration of 10 immune cells. Three subtypes showed a similar enrichment pattern of CD8 T cells with CIBERSORT and ssGSEA results, and other immune cells also differentially enriched in three subtypes, indicating that there was a huge difference of TME among them and thus resulted in discrepant prognosis.

**Figure 6:**
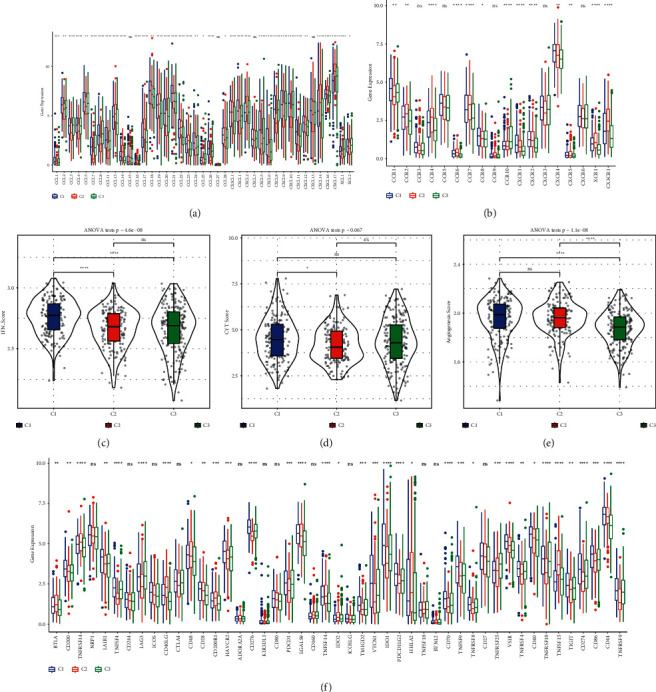
Characterizing the different TME among three subtypes. (a‒b) Gene expression of chemokines and chemokine receptors in three subtypes. (c‒e) Enrichment score of IFN-*γ*, CYT and angiogenesis in three subtypes. (f) The expression of 47 immune checkpoints in three subtypes. ANOVA test was conducted. NS, no significance. ^∗^*P* < 0.05, ^∗∗^*P* < 0.01, ^∗∗∗^*P* < 0.001, and ^∗∗∗∗^*P* < 0.0001.

Chemokines and chemokine receptors are a group of factors that orchestrate the migration or cell-cell signaling between different cell types, which are secreted by tumor cells or immune cells. We used ssGSEA to calculate their normalized gene expression in TCGA-LUAD dataset. A large fraction of chemokines (90.24%, 37 of 44) and chemokine receptors (72.22%, 13 of 28) manifested differential expression among three subtypes (*P* < 0.05, Figures 6(a) and 6(b)). It has been demonstrated that interferon-gamma (IFN-*γ*), cytolytic activity, and angiogenesis serve as indicative biomarkers in anti-tumor response and cancer progression [34–36].

Therefore, we searched a series of gene signatures of three features from previous studies [19–21], and characterized their enrichment score in three subtypes. The result showed that C1 subtype had the highest score of IFN-*γ* and C3 subtype manifested the lowest angiogenesis score, while no obvious difference of CYT score was shown among three subtypes (Figures 6(c)–6(e)). High expression of IFN-*γ* was reported to be associated with progressive tumor and worse prognosis by elevating IDO1 expression [37, 38]. It was sensible that C1 subtype with the highest IFN-*γ* score had unfavorable prognosis, although C1 presented a bit high CYT to against tumor cells. Moreover, we assessed the expression of 47 immune checkpoint obtained from Danilova et al. [19]. 35 of 47 immune checkpoints had the distinct expression among three subtypes (*P* < 0.05, Figure 6(f)), which may affect the efficacy of immunotherapy. Notably, IDO1 was the most expressed in C1 subtype and the least in C3 subtype, which was accordant with the abovementioned results.

### 3.5. C2 Subtype Is the Most Sensitive to Immunotherapy Predicted by TIDE Analysis

**Figure 7:**
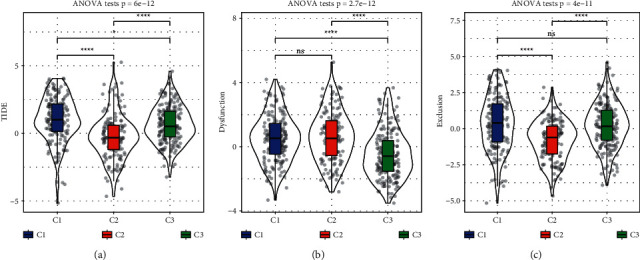
TIDE analysis for predicting the response to immunotherapy indicated by TIDE score (a), T cell dysfunction (b), and T cell exclusion (c). ANOVA test was conducted. NS, no significance. ^∗^*P* < 0.05 and ^∗∗∗∗^*P* < 0.0001.

As significantly differential TME was delineated in three subtypes, we then tried to understand their response to immune checkpoint blockade. TIDE analysis was implemented to calculate three scores including TIDE score, T cell dysfunction and T cell exclusion. As a result, C2 subtype had the lowest TIDE score among them, suggesting that C2 was predicted to be the most sensitive to immunotherapy (Figure 7(a)). Although T cell function was less damaged in C3 subtype, high T cell exclusion was present in C3 subtypes simultaneously (Figures 7(b) and 7(c)), which possibly lead to worse prognosis.

### 3.6. Establishing a Prognostic Model Based on Epi-PCGs

500 TCGA-LUAD samples were randomly divided into training and test groups with a ratio of 3 : 2. No significant difference was detected between two groups using Chi-square test (*P* > 0.05, Supplementary Table S2). In the training group, we applied univariate Cox regression analysis to screen 71 epi-PCGs from 122 epi-PCGs that were included in consensus clustering (*P* < 0.05). To reach an optimal model without too many prognostic genes, we performed LASSO Cox regression analysis to deduct the unnecessary genes. The coefficients of genes were close to zero with the increasing lambda (Supplementary Figure S4). When lambda = 0.0905, the optimal model was generated, and 10 epi-PCGs were remained. Then stepAIC was conducted to further decrease the number of genes and simplify the model. Finally, 5 epi-PCGs were determined to construct the prognostic model with coefficients from LASSO. The 5-gene prognostic model was defined as: risk score = 0.456^*∗*^LDHA + 0.278 ^*∗*^ EIF3B + 0.09^*∗*^TNS4 + 0.08^*∗*^LY6K − 0.385^*∗*^PDIK1L.

**Figure 8:**
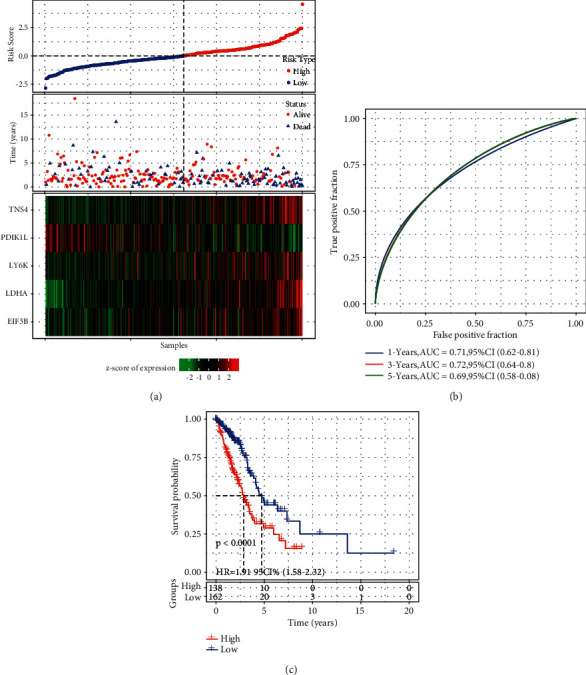
The performance of 5-gene prognostic model in the training group. (a) The survival and expression of each sample ranking by risk score. Horizontal axis represents samples. (b) ROC analysis for evaluating the efficiency in predicting 1-year, 3-year, and 5-year survival. (c) Kaplan‒Meier survival analysis for high-risk and low-risk groups. Log-rank test was conducted. HR, hazard ratio. AUC, area under ROC curve.

According to the prognostic model, we calculated the risk score for each sample in the training group. Samples were stratified into high-risk and low-risk groups by the cut-off of the z-score = 0 (Figure 8(a)). Obviously, samples with dead status were apparently enriched in high-risk group. Besides PDIK1L, other four epi-PCGs were relatively higher expressed in high-risk group (Figure 8(a)). ROC analysis revealed that the prognostic model had favorable performance in predicting 1-year, 3-year, and 5-year prognosis with AUC of 0.71, 0.72, and 0.69, respectively, (Figure 8(b)). High-risk group of 138 samples and low-risk group of 162 samples had a significant difference of overall survival (*P* < 0.0001, Figure 8(c)).

**Figure 9:**
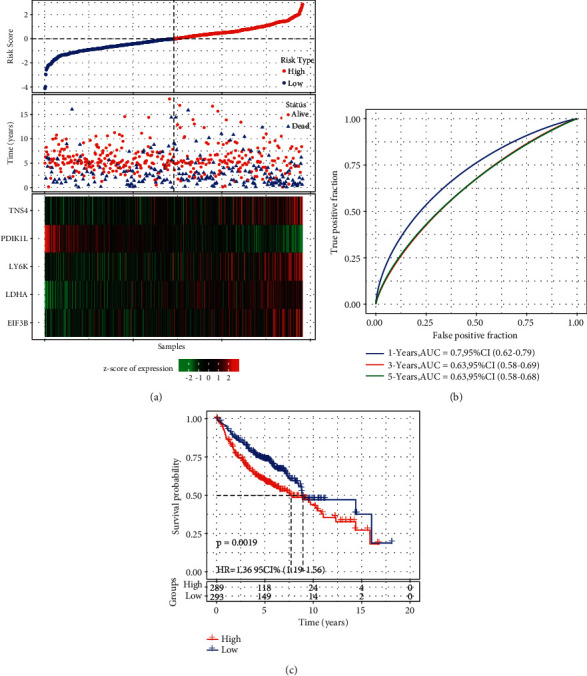
The performance of 5-gene prognostic model in the validation group. (a) The survival and expression of each sample ranking by risk score. Horizontal axis represents samples. (b) ROC analysis for evaluating the efficiency in predicting 1-year, 3-year, and 5-year survival. (c) Kaplan‒Meier survival analysis for high-risk and low-risk groups. Log-rank test was conducted. HR, hazard ratio. AUC, area under ROC curve.

In the test group, we performed the same analysis to confirm the ability of the prognostic model for predicting prognosis. A similar result was presented that high-risk group with 100 samples had worse survival than low-risk group with 100 samples (*P*=0.014, Supplementary Figure S5). Using all samples in TCGA-LUAD dataset, we delineated the distribution of their survival status and 5 gene expression ranking by risk score (Supplementary Figure S6). The prognostic model was able to stratify samples into high-risk and low-risk groups with distinct overall survival (*P* < 0.0001, Supplementary Figure S6). To verify the robustness of the model, GEO dataset was introduced as an independent validation group. A total of 289 and 293 samples were grouped into high-risk and low-risk groups, respectively, with differential overall survival (*P*=0.0019, Figure 9). Favorable AUC of 1-year, 3-year, and 5-year prognosis were shown with 0.79, 0.63, and 0.63, respectively. The abovementioned results validated the robust performance of the 5-gene prognostic model for predicting prognosis of LUAD patients.

### 3.7. Risk Score Is Differentially Distributed in Different Clinical Features

**Figure 10:**
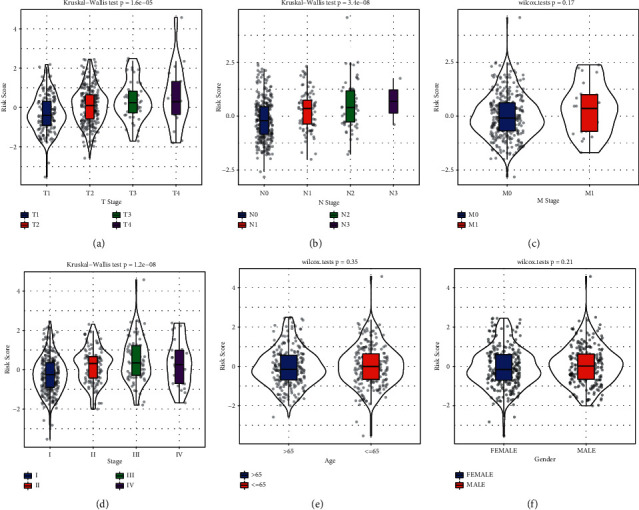
The distribution of risk score in different clinical features including T stage (a), N stage (b), M stage (c), stage (d), ages (e), and genders (f). Kruskal‒Wallis test was conducted among four groups and Wilcoxon test was conducted between two groups.

As risk score was proved to be significantly associated with prognosis, we next evaluated the relation between risk score and clinical features including TNM stage, ages and genders (Figure 10). It was obviously exhibited that with the increased severity of stages, the risk score elevated accordingly in *T* stage, N stage and stage (*P* < 0.0001, Figures 10(a) and 10(b) and 10(d)). A higher median risk score was observed in M1, but no difference was shown between M0 and M1 stages, which was possibly due to the small fraction of M1 samples (Figure 10(c)). In addition, ages and genders seemed not the factor resulting in the distribution of risk score (Figures 10(d) and 10(e)). We also examined the risk score in stratifying samples with different clinical features. Consequently, all samples with different clinical features could be stratified into high-risk and low-risk groups with discrepant overall survival (*P* < 0.05, Supplementary Figure S7).

### 3.8. Identifying Functional Pathways Associated with Risk Score

**Figure 11:**
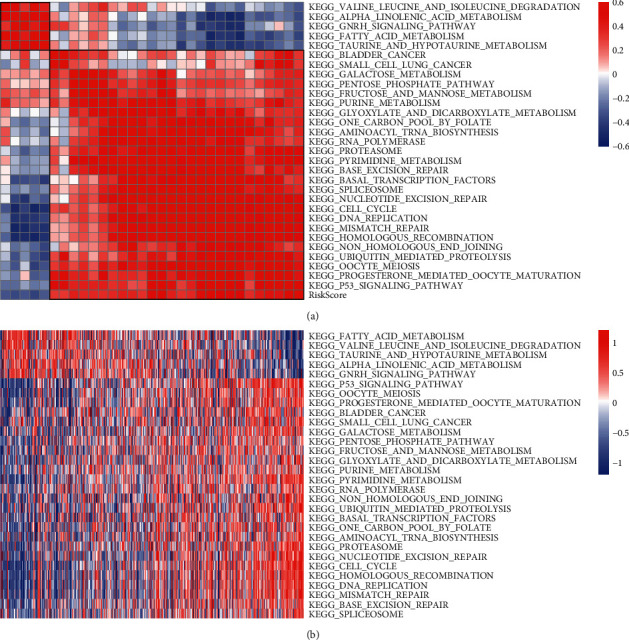
Identification of KEGG pathways highly associated with risk score. (a) Pearson correlation analysis between risk score and 30 KEGG pathways. Red means positive correlation and blue means negative correlation. (b) A heatmap of enrichment score of 30 KEGG pathways ranking by risk score (horizontal axis). Red indicates relatively high enrichment and blue indicates relatively low enrichment.

Risk score calculated based on epi-PCGs was able to distinguish high-risk patients, and a close association was also observed between risk score and stages. We suspected that these five epi-PCGs were highly involved in tumorigenesis or tumor progression. Therefore, to understand which functional pathways these epi-PCGs were enriched, we employed ssGSEA to calculate the enrichment score of KEGG pathways for each sample in TCGA-LUAD dataset. Then Pearson correlation analysis was used to screen correlative KEGG pathways with risk score (|correlation coefficient| > 0.3). The top 30 KEGG pathways were visualized with 25 positively 5 negatively related to risk score (Figure 11). A fraction of tumor-related pathways were highly enriched, such as bladder cancer, small cell lung cancer, and p53 signaling pathway. A number of cell proliferation-related pathways such as cell cycle, DNA replication, and mismatch repair were identified. Furthermore, we used the same method to analyze the enrichment scores of Wiki pathways and their relationships to risk score. Similar results were outputted that cell cycle, DNA repair-related pathways and tumor-related pathways were also significantly enriched (Supplementary Figure S8A). These pathways were positively correlated with risk score (Supplementary Figure S8B). These enriched pathways indirectly supported that dysregulated histone modifications on these epi-PCGs made a strong effect on a series of oncogenic pathways that were involved in tumorigenesis.

### 3.9. Constructing a Nomogram Based on Risk Score for Application in Clinical Management

**Figure 12:**
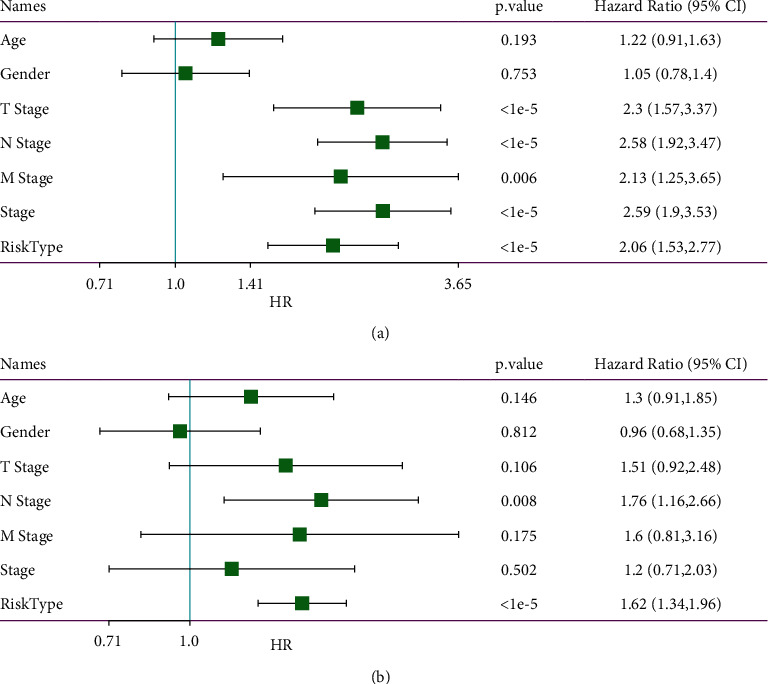
Univariate (a) and multivariate (b) Cox regression analysis of risk score and clinical features in the relation to prognosis. Log-rank test was conducted. HR, hazard ratio. CI, confidence interval.

**Figure 13:**
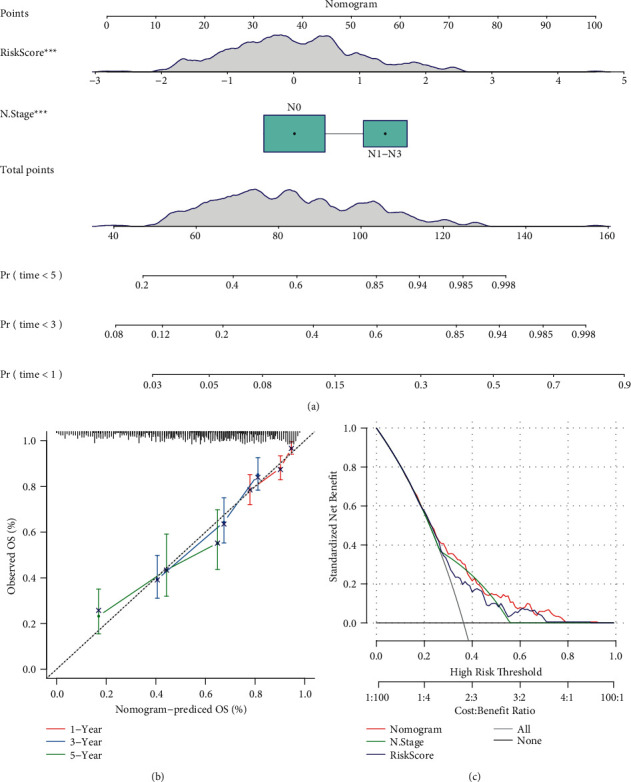
Establishing a nomogram based on risk score. (a) A nomogram based on risk score and N stage for predicting 1-year, 3-year, and 5-year death rate. (b) Calibration curve of 1-year, 3-year, and 5-year overall survival predicted by the nomogram. (c) DCA curve of the nomogram, N stage and risk score. OS, overall survival.

Given that the satisfactory performance of the 5-gene prognostic model, we attempted construct a nomogram based on the risk score. Before that, univariate and multivariate Cox regression analysis were conducted to assess the independence of the prognostic model (named as risk type in Figure 12) together with other clinical features. The result manifested that N stage and risk type were both the independent risk factor of LUAD, with HR = 1.76 (95%CI = 1.16–2.66) and HR = 1.62 (95%CI = 1.31–1.96), respectively, in multivariate analysis (Figure 12). Then we used N stage and risk score to construct the nomogram based on samples in TCGA-LUAD dataset. A patient could obtain a total points according to risk score and N stage, and the corresponding 1-year, 3-year, and 5-year death rates were indicated in the nomogram (Figure 13(a)). The comparison between predicted overall survival and observed overall survival shown the reliability of the nomogram (Figure 13(b)). Moreover, DCA was performed to evaluate the effectiveness and benefit that LUAD patients could obtain from the nomogram, risk score, and N stage (Figure 13(c)). It could be concluded that LUAD patients could reach the optimal outcome using the least cost with the assistant of the nomogram in clinical.

### 3.10. Comparison with Other Prognostic Models of LUAD

**Figure 14:**
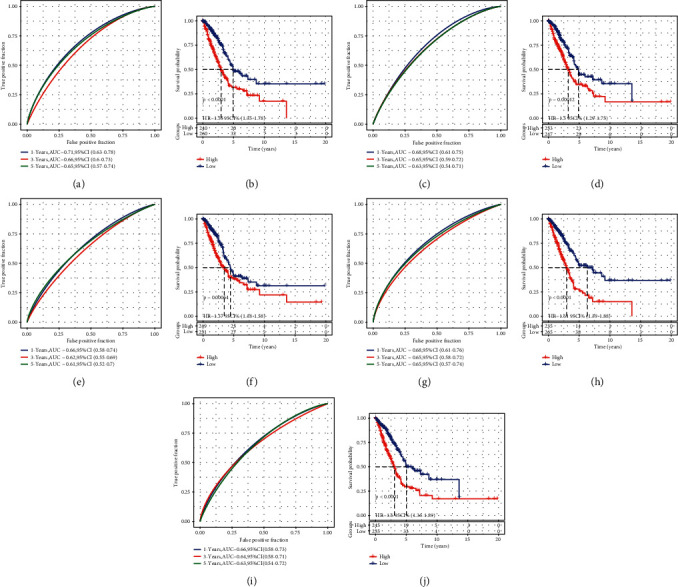
Comparison with other gene signatures of LUAD obtained from other studies including Li et al. (a‒b), Xue et al. (c‒d), Liu et al. (e‒f), Sun et al. (g‒h), and Al-Dherasi et al. (i‒j). A, C, E, G, and J represent ROC curves and B, D, F, H, and J represents Kaplan‒Meier survival plots. Log-rank test was conducted.

Given that other studies have also explored a series of prognostic signatures for LUAD, we included some signatures from the following five studies including Li et al. [30], Xue et al. [33], Liu et al. [31], Sun et al. [32], and Al-Dherasi et al. [29], and used the same method in our study to fairly compare the ability of these signatures to ours. By using the same dataset of TCGA-LUAD, five prognostic signatures were all efficient to classify samples into high-risk and low-risk groups with favorable AUC score and distinct survival as well (*P* < 0.0001, Figure 14). However, our 5-gene signature still performed the highest AUC of 1-year and 3-year survival prediction with 0.74 (95%CI = 0.67–0.81) and 0.70 (95%CI = 0.64–0.77), respectively, (Supplementary Figure S6). Although an 8-gene signature from Li et al. and a 4-gene signature from Sun et al. had a bit higher AUC than our 5-gene signature (Supplementary Figures S6A and S6G), their risk score had a lower HR than ours (HR = 1.84, 95%CI = 1.57–2.15). Overall, our 5-gene signature showed the best performance in predicting LUAD prognosis in TCGA-LUAD dataset.

## 4. Discussion

Extensive studies have demonstrated the important role of histone modifications on DNA transcription machinery and accessibility of other regulators [39]. Not surprisingly, the alternations of histone modification can affect the expression or post-translational modification of genes associated with cancer development. We investigated the state of histone modifications locating enhancers and promoters in LUAD, and observed distinct expression of the genes with dysregulated histone modifications between normal tissues and tumor tissues, suggesting that histone modifications especially in promoters were involved in tumorigenesis through regulating expression level.

H3K9me3 and H3K27me3 modifications seem to have a protective effect that significantly higher rate of modifications were shown in normal tissues. Abundant evidences have shown that the aberrant H3K9me3 modification is associated with tumorigenesis, but may vary in different cancer types. The methyltransferase of H3K9me3, SUV39H1, was up-regulated in colorectal cancer cells, and cancer cell migration was inhibited by the knockdown of SUV39H1 [40]. In breast cancer, Li et al. demonstrated that reduced H3K9me3 lead to the increased sensitivity to DNA damage and thus result in breast cancer transformation [41]. High expression level of H3K9 demethylases such as LSD1 or JMJD2C promoted melanomagenesis, and targeted inhibition on the demethylases restored immune response and controlled tumor cell growth [42]. In the mouse model of lung cancer, LSD1 inhibitor (SP2509) increased H3K9me3 level and predominantly inhibit the proliferation of lung cancer cells with menin-low expression, supporting that decreased H3K9me3 was a risk factor in lung cancer [43]. Ávila‒Moreno et al. found decreased level of H3K9me3 in the promoter of MEOX2 and TWIST1, whose overexpression was associated with poor prognosis in lung cancer [44]. High expression of H3K9me3 was considered as an indicator to represent better prognosis for NSCLC patients [45], which was consistent with our result.

The levels of H3K27ac and H3K4me3 were opposite in enhancers and promoters compared with normal ones, with both of them had decreased levels in promoters, indicating that they served as different mechanisms in regulating gene expression. In the relation between different modifications and pathways, tumor-related pathways and immune-related pathways were highly enriched, which supported the critical role of these modifications in tumorigenesis and anti-tumor immune response. According to the abovementioned observations, we constructed a molecular subtyping system based on epi-PCGs.

Three molecular subtypes had distinct prognosis with the best in C2 subtype and the worst in C3 subtype. Importantly, differential TME was shown among three subtypes, which lead to discrepant survival and response to immunotherapy to large extent. The enrichment of CD8 T cells was higher in C1 and C3 subtypes, which may lead to favorable immune response theoretically. However, an extremely higher enrichment of M0 macrophages was also presented in the two subtypes, where M0 was reported to be associated with unfavorable prognosis [46–48]. The role of macrophages in stromal and tumor is complicated, which is modulated by various factors such as cytokines and chemokines. Overall, different TME and immune response among three subtypes suggested that histone modifications did a nonnegligible effect on prognosis, but needing further demonstration to verify their specific roles and mechanisms in tumorigenesis.

In addition, as the important role of epi-PCGs was illustrated, we further established a prognostic model based on epi-PCGs. Five genes were screened as prognostic genes, including LDHA, EIF3B, TNS4, LY6K, and PDIK1L. Besides PDIK1L playing a protective role, the expression of other four genes were all negatively correlated with poor survival. Human lactate dehydrogenase A (LDHA) was reported to be negatively associated prognosis that high LDHA expression predicted poor prognosis in LUAD [49]. Ooi et al. found that disrupting LDHA showed synergistic anti-tumor effects when combining with other chemotherapeutic drugs [50]. Eukaryotic initiation factor 3b (eIF3b) was discovered to promote tumor cell proliferation and progression in NSCLC [51], and also correlated with advanced stages in bladder and prostate cancer [52]. Lymphocyte antigen 6 complex locus K (LY6K) was considered as a molecular target to treat bladder cancer [53].

The 5-gene signature was robust to classify LUAD patients into high-risk and low-risk groups, and was highly associated with stage progression. Notably, oncogenic pathways such as p53 signaling, cell cycle, and DNA repair were massively enriched in LUAD patients with high-risk scores. These five prognostic genes may serve as molecular targets for exploring new therapeutic drugs and tumorigenesis mechanisms in LUAD. In addition, compared with other prognostic models in the previous research, our 5-gene signature exhibited the most favorable performance in predicting prognosis. Notably, to our knowledge, no research has proposed molecular subtypes or prognostic signatures based on histone modifications for LUAD till now. However, this study only based on pure bioinformatics analysis, the signature based on epigenetic modifications needs to be further verified in more clinical samples.

In conclusion, we established three novel molecular subtypes and a 5-gene signature based on epi-PCGs with dysregulated histone modifications. We further illustrated the nonnegligible role of histone modifications in orchestrating different TME contributing for different immune response. These novel molecular subtypes and the prognostic model could provide a guidance in assisting decision-makings for LUAD treatment.

## Data Availability

The dataset used in this study is available in GSE19188 [https://www.ncbi.nlm.nih.gov/geo/query/acc.cgi?acc=GSE19188], in GSE30219 [https://www.ncbi.nlm.nih.gov/geo/query/acc.cgi?acc=GSE30219], in GSE31210 [https://www.ncbi.nlm.nih.gov/geo/query/acc.cgi?acc=GSE31210]; in GSE37745 [https://www.ncbi.nlm.nih.gov/geo/query/acc.cgi?acc=GSE37745], and in GSE50081[https://www.ncbi.nlm.nih.gov/geo/query/acc.cgi?acc=GSE50081].

## Supplementary Materials

Supplementary Materials Supplementary Figure S1. Preprocessing the gene expression data of GSE cohorts. PCA plots before (A) and after (B) removing batch effects. PCA, principle component analysis. Supplementary Figure S2. Annotation of GO terms and KEGG pathways by clusterProfiler. Size means the number of genes enriched in one term. Supplementary Figure S3. The expression of 122 epi-PCGs in normal and tumor samples. TPM, transcript per million. Student's *t*-test was conducted. ^∗∗∗^*P* < 0.001 and ^∗∗∗∗^*P* < 0.0001. Supplementary Figure S4. LASSO Cox regression analysis for optimizing the prognostic model. (A) The trajectory of coefficients varied by the increasing lambda. (B) Partial likelihood deviance of each lambda. Red dotted line and red dot indicate lambda = 0.0485. Supplementary Figure S5. The performance of 5-gene prognostic model in the test group. (A) The survival and expression of each sample ranking by risk score. Horizontal axis represents samples. (B) ROC analysis for evaluating the efficiency in predicting 1-year, 3-year, and 5-year survival. (C) Kaplan‒Meier survival analysis for high-risk and low-risk groups. Log-rank test was conducted. HR, hazard ratio. AUC, area under ROC curve. Supplementary Figure S6. The performance of 5-gene prognostic model in TCGA-LUAD dataset. (A) The survival and expression of each sample ranking by risk score. Horizontal axis represents samples. (B) ROC analysis for evaluating the efficiency in predicting 1-year, 3-year, and 5-year survival. (C) Kaplan‒Meier survival analysis for high-risk and low-risk groups. Log-rank test was conducted. HR, hazard ratio. AUC, area under ROC curve. Supplementary Figure S7. Kaplan‒Meier survival plots of high-risk and low-risk groups with different clinical features. Log-rank test was conducted. Supplementary Figure S8. (A) A heatmap of significantly enriched Wiki pathways in TCGA dataset. Horizontal axis indicates the samples and the vertical axis indicates the enriched pathways. The z-score of ssGSEA was used. (B) The correlation analysis between risk score and enriched Wiki pathways. Red and blue indicate positive and negative correlations, respectively. Supplementary Table S1. Clinical information of TCGA-LUAD and GEO datasets. Supplementary Table S2. Clinical information of training and test groups.
