# Supplementary material for: Establishing a Novel Gene Signature Related to Histone Modifications for Predicting Prognosis in Lung Adenocarcinoma
Source: J Oncol. 2022 Sep 23;2022:8802573. doi: 10.1155/2022/8802573 (PMC9525801; doi:10.1155/2022/8802573)
Supplement: Supplementary Materials — Supplementary Figure S1. Preprocessing the gene expression data of GSE cohorts. PCA plots before (A) and after (B) removing batch effects. PCA, principle component analysis. Supplementary Figure S2. Annotation of GO terms and KEGG pathways by clusterProfiler. Size means the number of genes enriched in one term. Supplementary Figure S3. The expression of 122 epi-PCGs in normal and tumor samples. TPM, transcript per million. Student's t-test was conducted. ∗∗∗P < 0.001 and ∗∗∗∗P < 0.0001. Supplementary Figure S4. LASSO Cox regression analysis for optimizing the prognostic model. (A) The trajectory of coefficients varied by the increasing lambda. (B) Partial likelihood deviance of each lambda. Red dotted line and red dot indicate lambda = 0.0485. Supplementary Figure S5. The performance of 5-gene prognostic model in the test group. (A) The survival and expression of each sample ranking by risk score. Horizontal axis represents samples. (B) ROC analysis for evaluating the efficiency in predicting 1-year, 3-year, and 5-year survival. (C) Kaplan‒Meier survival analysis for high-risk and low-risk groups. Log-rank test was conducted. HR, hazard ratio. AUC, area under ROC curve. Supplementary Figure S6. The performance of 5-gene prognostic model in TCGA-LUAD dataset. (A) The survival and expression of each sample ranking by risk score. Horizontal axis represents samples. (B) ROC analysis for evaluating the efficiency in predicting 1-year, 3-year, and 5-year survival. (C) Kaplan‒Meier survival analysis for high-risk and low-risk groups. Log-rank test was conducted. HR, hazard ratio. AUC, area under ROC curve. Supplementary Figure S7. Kaplan‒Meier survival plots of high-risk and low-risk groups with different clinical features. Log-rank test was conducted. Supplementary Figure S8. (A) A heatmap of significantly enriched Wiki pathways in TCGA dataset. Horizontal axis indicates the samples and the vertical axis indicates the enriched pathways. The z-score of ssGSEA was used. (B) [file 8802573.f1.zip › Supplementary Table S1.docx]

Supplementary Table S1. Clinical information of TCGA-LUAD and GEO datasets.

| **Clinical Features** | **TCGA-LUAD** | **GSE19188** | **GSE30219** | **GSE37745** | **GSE50081** | **GSE31210** |
| --- | --- | --- | --- | --- | --- | --- |
| **OS** |  |  |  |  |  |  |
| 0 | 318 | 16 | 40 | 29 | 76 | 191 |
| 1 | 182 | 24 | 43 | 77 | 51 | 35 |
| **T Stage** |  |  |  |  |  |  |
| T1 | 167 |  | 69 |  | 43 | 168 |
| T2 | 267 |  | 12 |  | 82 | 58 |
| T3 | 45 |  | 2 |  | 2 |  |
| T4 | 18 |  |  |  |  |  |
| TX | 3 |  |  |  |  |  |
| **N Stage** |  |  |  |  |  |  |
| N0 | 324 |  | 80 |  | 94 |  |
| N1 | 94 |  | 3 |  | 33 |  |
| N2 | 69 |  |  |  |  |  |
| N3 | 2 |  |  |  |  |  |
| NX | 11 |  |  |  |  |  |
| **M Stage** |  |  |  |  |  |  |
| M0 | 332 |  | 83 |  | 127 |  |
| M1 | 24 |  |  |  |  |  |
| MX | 144 |  |  |  |  |  |
| **Stage** |  |  |  |  |  |  |
| I | 268 |  |  | 70 | 92 |  |
| II | 119 |  |  | 19 | 35 |  |
| III | 80 |  |  | 13 |  |  |
| IV | 25 |  |  | 4 |  |  |
| X | 8 |  |  |  |  |  |
| **Gender** |  |  |  |  |  |  |
| Female | 270 | 15 | 18 | 60 |  | 121 |
| Male | 230 | 25 | 65 | 46 |  | 105 |
| **Age** |  |  |  |  |  |  |
| <=65 | 237 |  | 60 | 57 | 40 |  |
| >65 | 253 |  | 23 | 49 | 87 |  |
| NA | 10 |  |  |  |  |  |
